# Supplementary material for: The importance of study design for detecting differentially abundant features in high-throughput experiments
Source: Genome Biol. 2014 Dec 3;15(12):527. doi: 10.1186/s13059-014-0527-7 (PMC4253014; doi:10.1186/s13059-014-0527-7)
Supplement: Additional file 1: — Supplementary Figures and Tables. [file 13059_2014_527_MOESM1_ESM.pdf]

The importance of study design for detecting differentially abundant features in high-throughput experiments

Luo Huaien[1*], Li Juntao[1*], Chia Kuan Hui Burton[1], Paul Robson[2], Niranjan Nagarajan[1#]

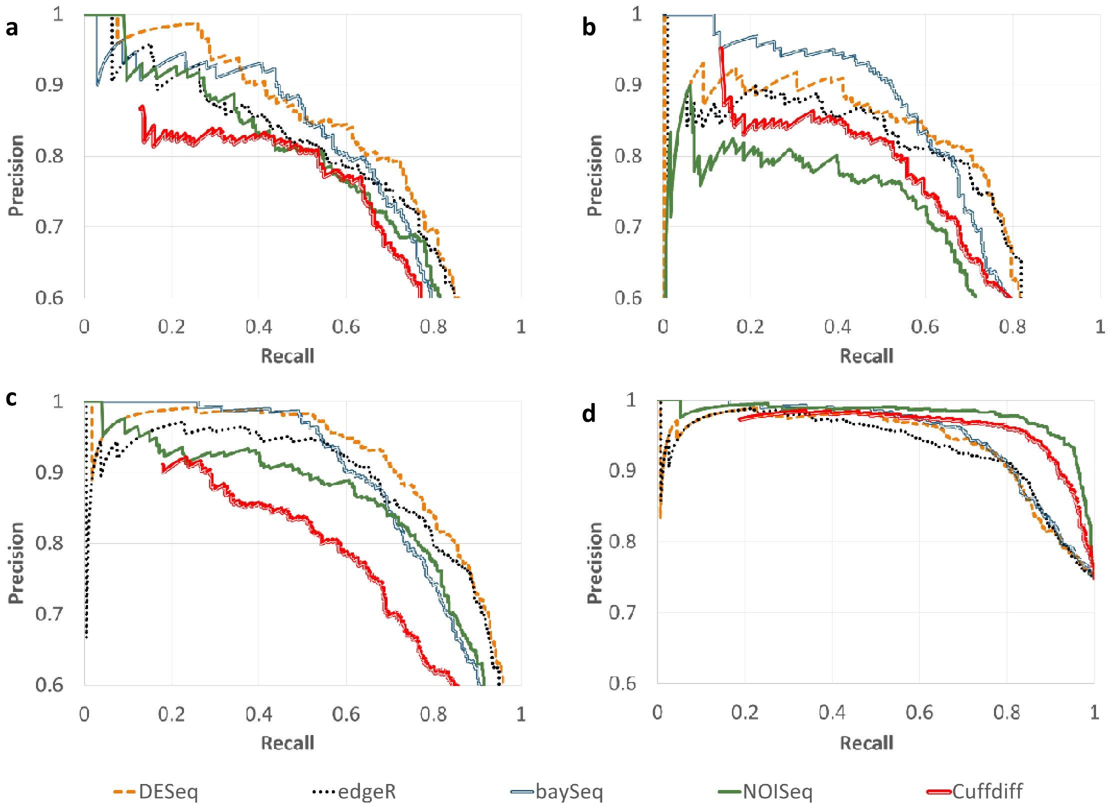

Supplementary Figure 1. Variability in performance across DATs with few replicates. The subfigures depict precision-recall tradeoffs for DATs, under a range of experimental conditions and with few replicates (2 in this case). The following parameters were used in EDDA for the subfigures: (a) PDA=30% and FC=Uniform[2,6] (b) as in subfigure (a) but with AP=HBR (c) PDA=[15% UP, 30% DOWN] and FC=Uniform[3,15] (d) PDA=[50% UP, 25% DOWN], FC=Normal[5,1.33] and SM=Full. Common parameters unless otherwise stated include AP=BP, EC=1000, ND=500 per entity, SM=NB and SV=0.5.

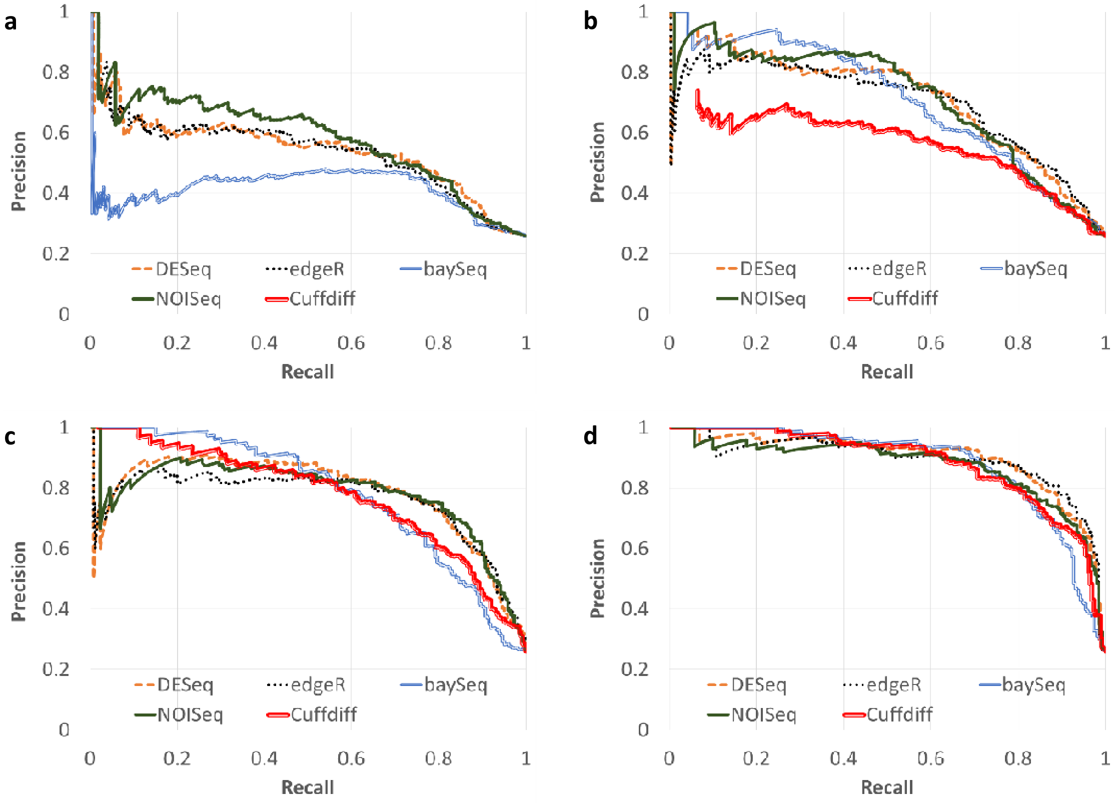

Supplementary Figure 2. Variability in performance across DATs with few data-points. Note that as in **Supplementary Figure 1**, the performance of DATs varies widely merely by changing the number of replicates from 1 to 4 in subfigures (a)-(d) in a situation where the number data-points generated is limited (only 50 on average per entity). Results reported are based on data generated in EDDA with the following parameters (roughly mimicking a microbial RNA-seq application): EC=1000, PDA=26%, FC=Uniform[3,7], AP=BP, SM=Full and SV=0.85.

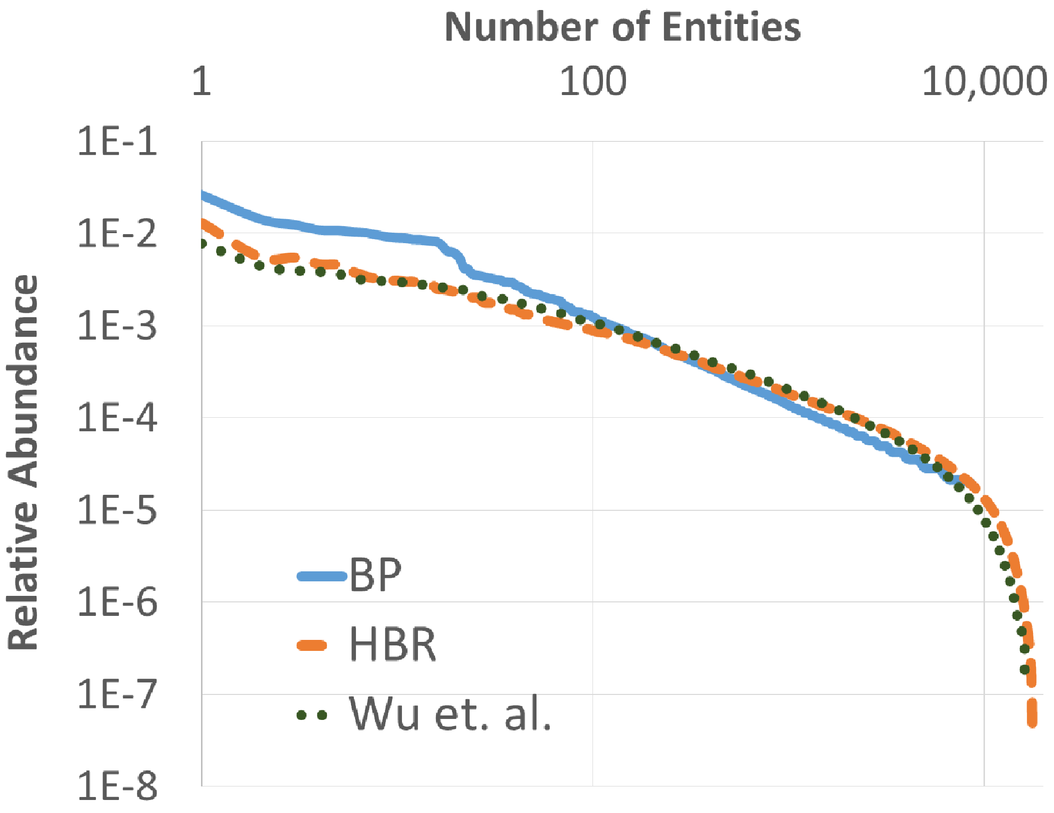

Supplementary Figure 3. Distribution of relative abundance in different empirically derived profiles. Note that both axes are plotted on a log-scale and that while BP does not have entities with relative abundance < 1e-5, the profile from Wu et al is more enriched for entities with relative abundance < 1e-5 than the HBR profile.

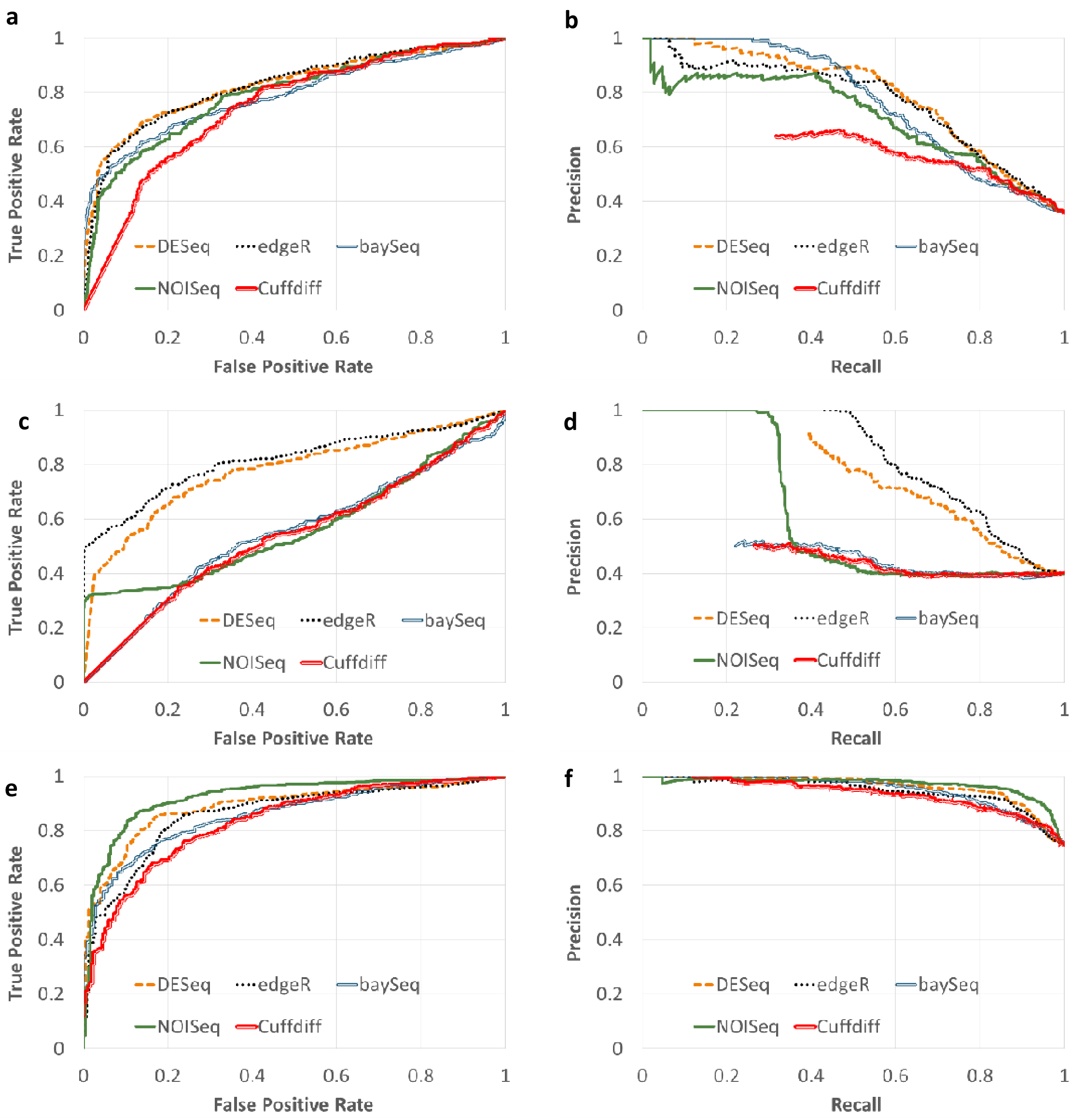

Supplementary Figure 4. Variability in performance of DATs under different perturbation profiles. The subfigures depict performance tradeoffs (TPR vs FPR and precision vs recall) under three different perturbation profiles and serve to highlight that different statistical tests are optimal under different experimental conditions. Results reported are based on the perturbation profiles and settings used in **Figure 5a** [subfigure (a) and (b)], **Figure 5c** [subfigure (c) and (d)] and **Figure 5d** [subfigure (e) and (f)].

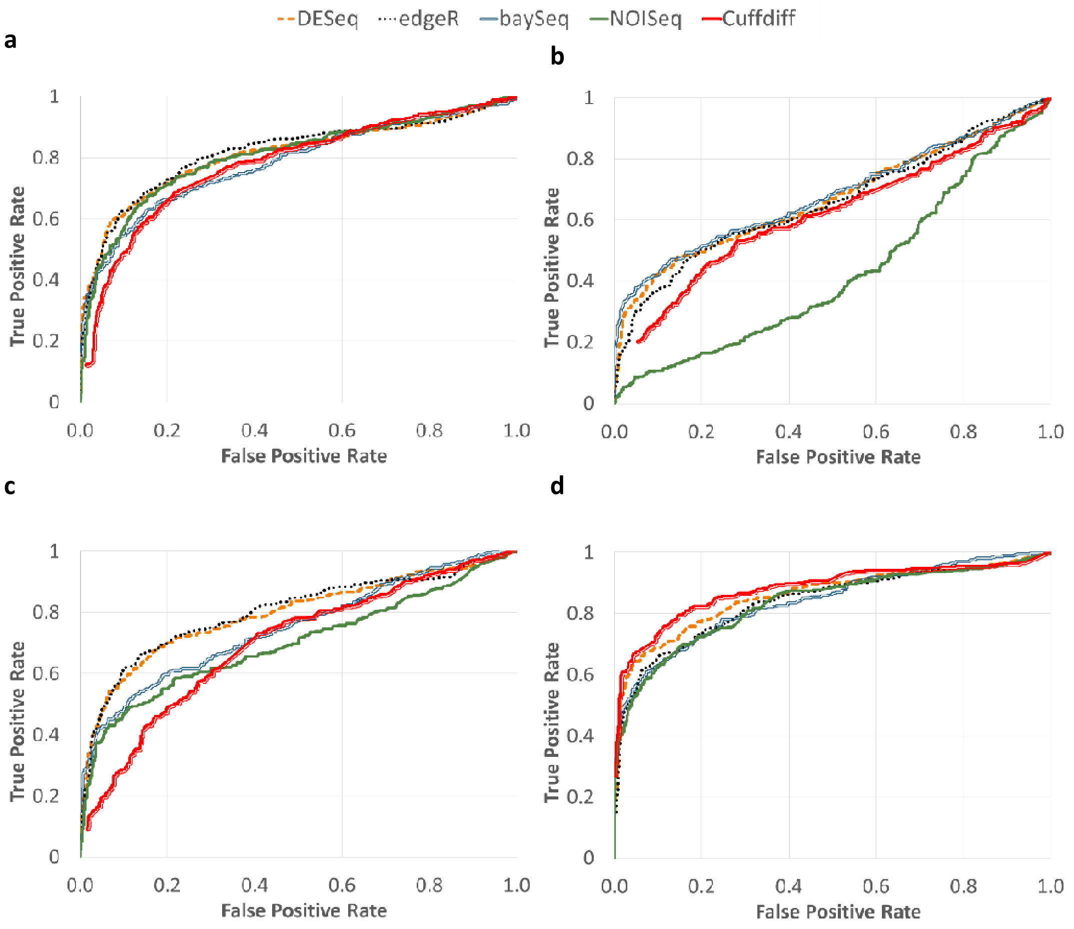

Supplementary Figure 5. Variability in performance as a function of the simulation model. As shown in subfigure (a), all DATs perform reasonably well under a Negative Binomial model in this setting. Switching to the Multinomial model as shown in subfigure (b) however seems to elicit worse performance in all DATs, even though in principle sample variability is not modelled in this setting making the dataset in some sense easier to call differentially abundant entities from. The joint Full model also seems to elicit a drop in performance for some DATs [subfigure (c)]. Finally subfigure (d) shows that improved performance can be obtained for all DATs when the data from subfigure c is mode-normalized. The common settings used in EDDA to generated these datasets are NR=2, EC=1000, PDA=[29% UP, 10% DOWN], FC=Log-normal[1.5, 1], ND=200 per entity, AP=HBR and SV=0.5.

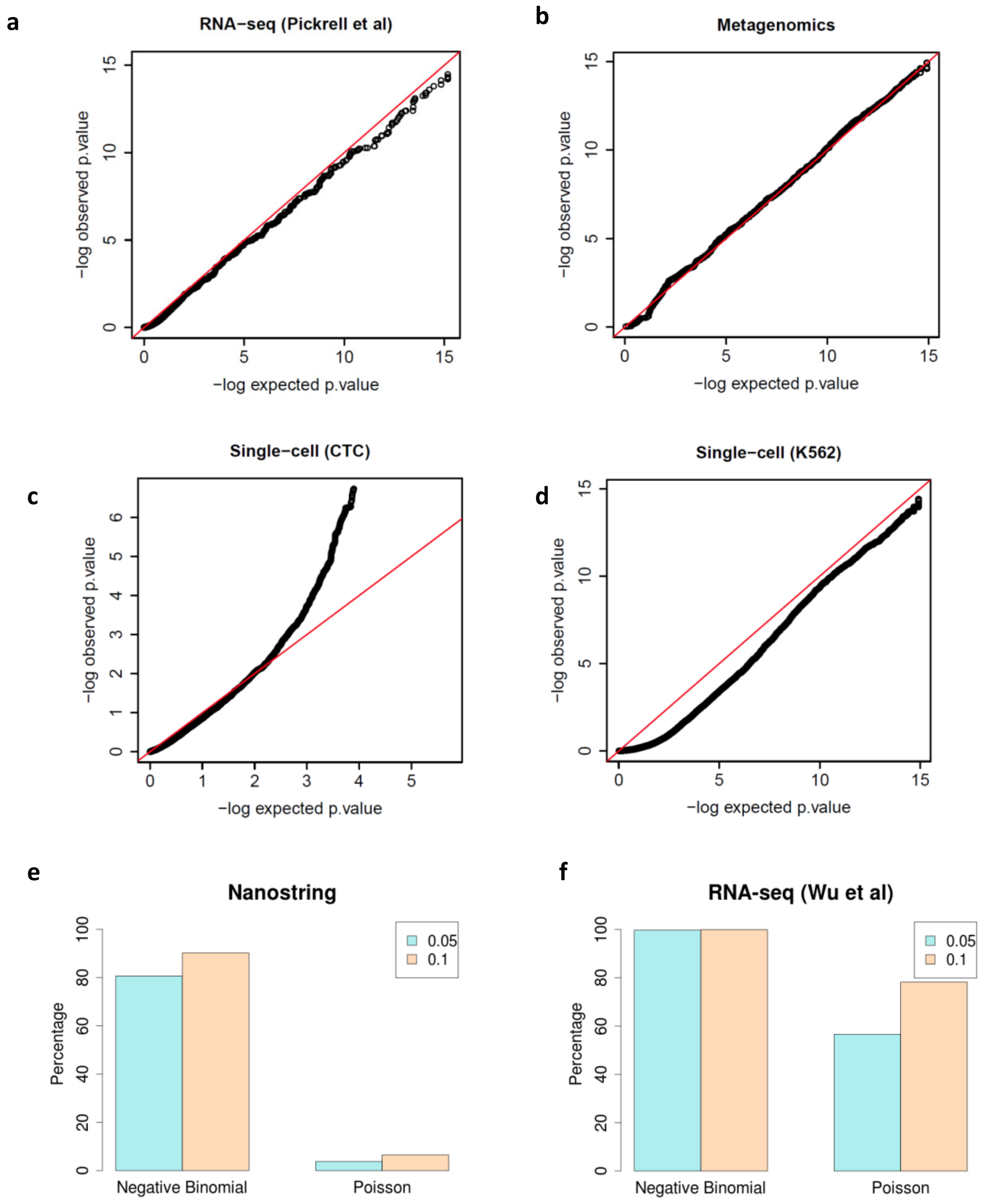

Supplementary Figure 6. Modelling sample variability using the Negative Binomial model. Subfigures (a) - (d) depict observed vs expected (based on simulated counts from a Negative Binomial model) *p*-values for each gene using the Kolmogorov-Smirnov (K-S) test to assess fit to a Negative Binomial distribution (using edgeR estimated parameters). (a) RNA-seq count data is well-approximated by a Negative Binomial model (data from Pickrell et al) (b) Metagenomic count data is well-approximated by a Negative Binomial model (data from Qin et al) (c, d) Single-cell RNA-seq data is not well-approximated (CTC single-cell data in Ramskold et al and K562 single-cell data). Subfigures (e) and (f) show the fraction of entities for which the fit to the Negative Binomial distribution is not rejected by a K-S test at two different significance thresholds (0.05 and 0.1), with results for the Poisson distribution provided for perspective. (e) Results for the Nanostring dataset used in this study (f) Results for bulk RNA-seq data from ENCODE (GM12892).

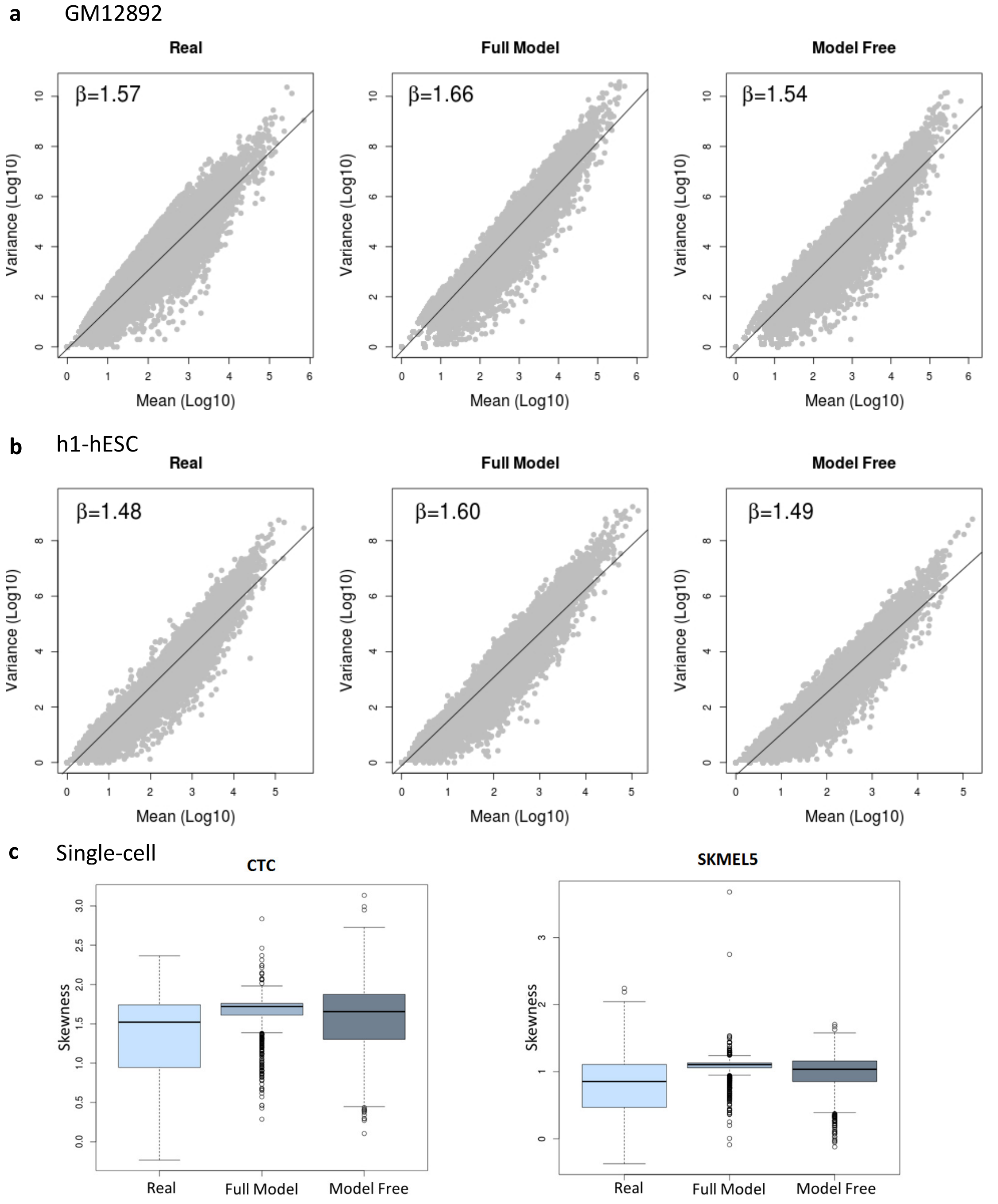

Supplementary Figure 7. Comparison of distributional properties for real datasets and EDDA simulations that mimic them. Results shown are for two ENCODE RNA-seq datasets (a) GM12892 and (b) h1-hESC and depict variance-mean relationship for original data and simulated counts from EDDA under the Full model and the Model-free approach. As seen here, the regression coefficients (β) are quite similar, demonstrating that the models do capture sample variability in real data. Linear regression line is for the model log(variance) ~ n*log(mean), where n > 1. (c) Boxplot of skewness of single-cell RNA-seq (Ramskold et al) and simulated counts from EDDA under the Full model and Model-free approaches. Note that the Model-free approach does better at capturing the variability in skewness.

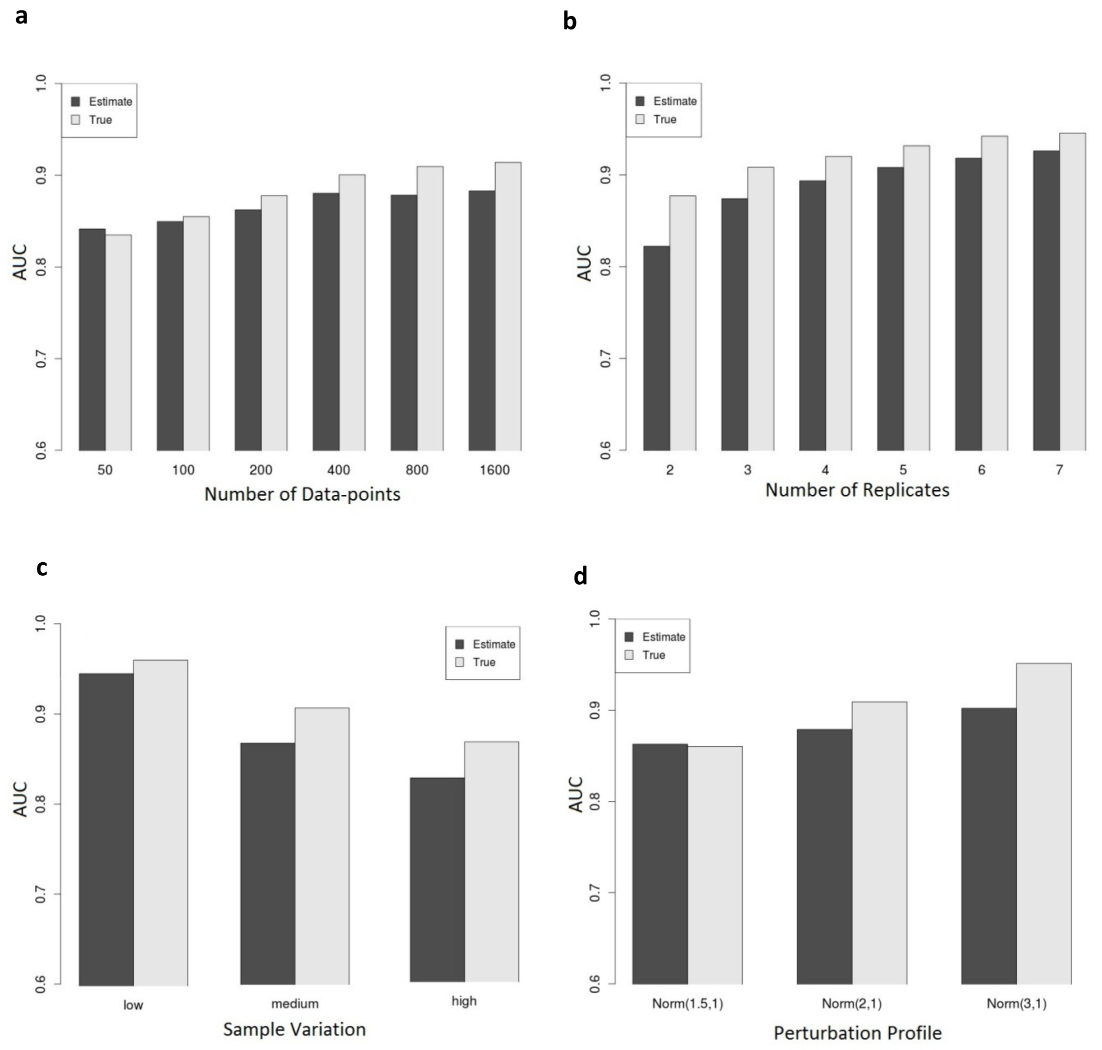

Supplementary Figure 8. EDDA simulations capture performance trends using simulated data. AUC values presented here were obtained from simulated datasets in EDDA (using edgeR analysis), generated by learning parameters from the input data. This approach was tested under different experimental settings: (a) varying number of data-points (ND=50, 100, 200, 400, 800, 1600); (b) varying number of replicates (NR=2, 3, 4, 5, 6, 7); (c) different sample variability (SV=0.1 [low], 0.5 [medium], 0.85 [high]) and (d) different perturbation profiles (Log-normal[1.5, 1], Log-normal[2, 1] and Log-normal[3, 1]). True AUC values were computed using edgeR on the input data (which were obtained from simulations with the Full model). Common parameters for all experiments include NR=3, EC=10000, PDA=20%, ND=200 per entity, AP=HBR, SM=Full.

a

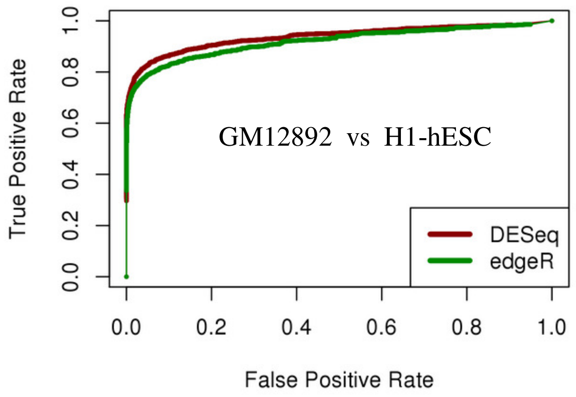

	edgeR	DESeq
AUC predicted by EDDA	0.91	0.94
Published Benchmarking Results	DESeq reports fewer false positives with similar sensitivity as edgeR	

b

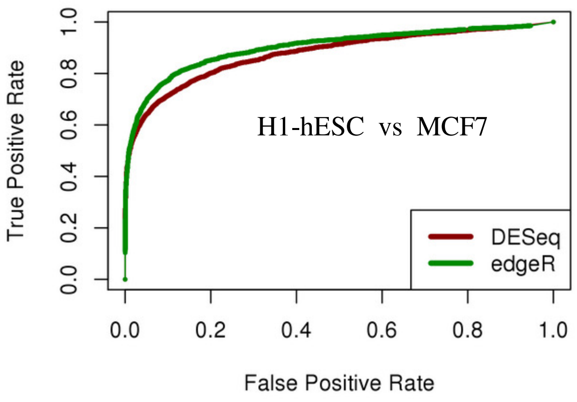

	edgeR	DESeq
AUC predicted by EDDA	0.90	0.87
Published Benchmarking Results	edgeR reports fewer false positives with similar sensitivity as DESeq	

c

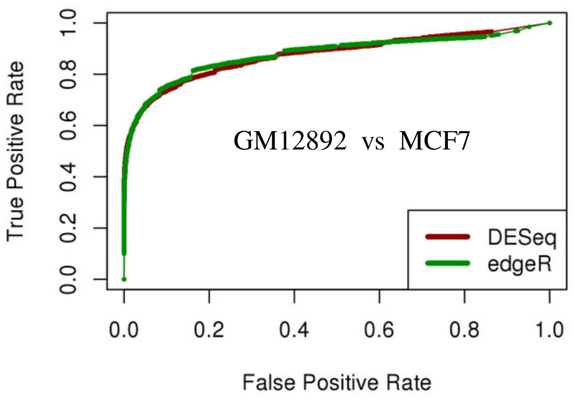

	edgeR	DESeq
AUC predicted by EDDA	0.88	0.88
Published Benchmarking Results	DESeq and edgeR have similar false positive rate and sensitivity	

Supplementary Figure 9. Performance evaluation of edgeR and DESeq for three RNA-seq datasets using EDDA agrees with published benchmark-based results[26]. (left) ROC curves from EDDA's performance evaluation module: (a) GM12892 vs H1-hESC (b) H1-hESC vs MCF7 (c) GM12892 vs MCF7. (right) Summary of AUC values predicted by EDDA and published benchmarking results[26].

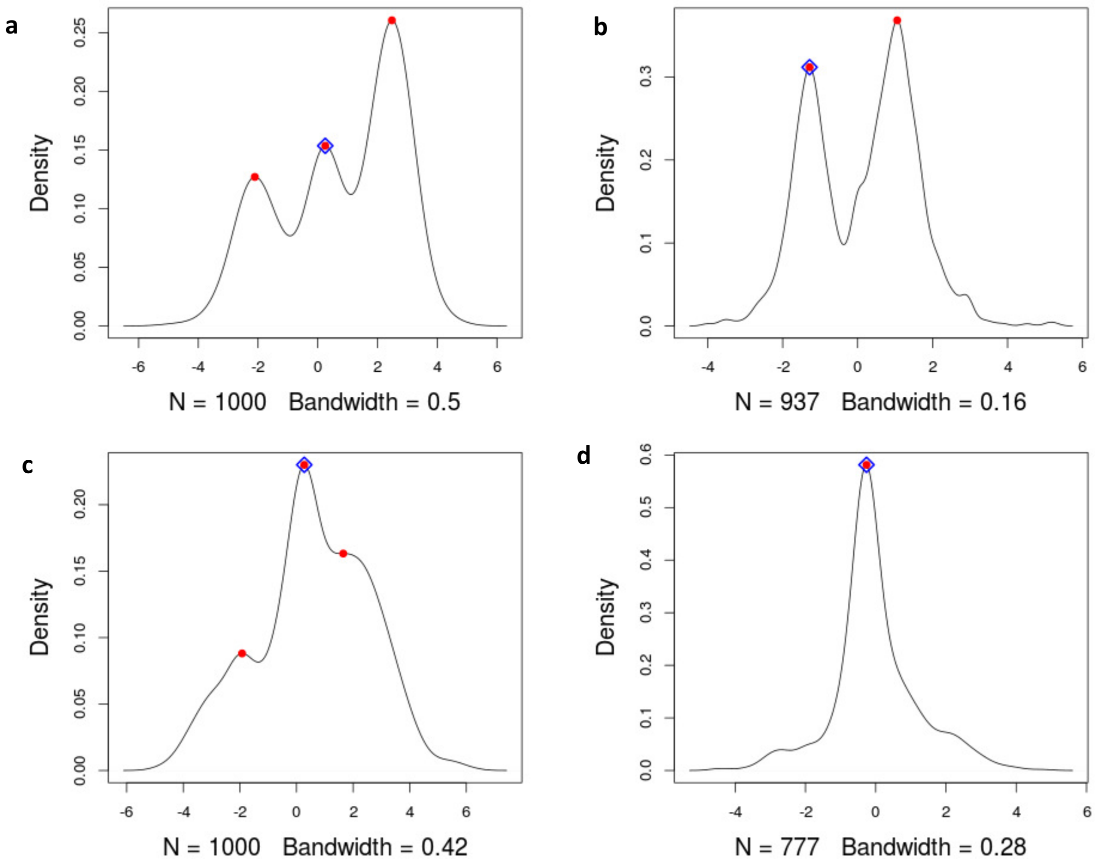

Supplementary Figure 10. Mode-normalization in practice. The various subfigures show the density plot for un-normalized fold changes (UFCs) under representative experimental settings. Note that empirical UFCs were log2 transformed and the smoothed distributed was obtained using a kernel density estimation approach (parameters used are on the x-axis). The identified modes are marked with red dots and the mode selected by EDDA is marked with a blue diamond, correctly identifying a set of non-differentially-abundant entities in all settings. The settings in EDDA for each of the subfigures were as follows: (b) NR=5, PDA=[60% UP], AP=HBR and SV=0.85 (c) PDA=[50% UP, 25% DOWN], FC=Log-normal[2, 1] (d) NR=5, PDA=[26% UP, 10% DOWN], FC=Log-normal[1.5, 1], AP=Wu et al, and SM=NB. Common parameters (unless otherwise stated) include NR=3, EC=1000, ND=500 per entity, PDA=[50% UP, 25% DOWN], FC=Uniform[3,7], AP=BP, SM=Full and SV=0.5.

a

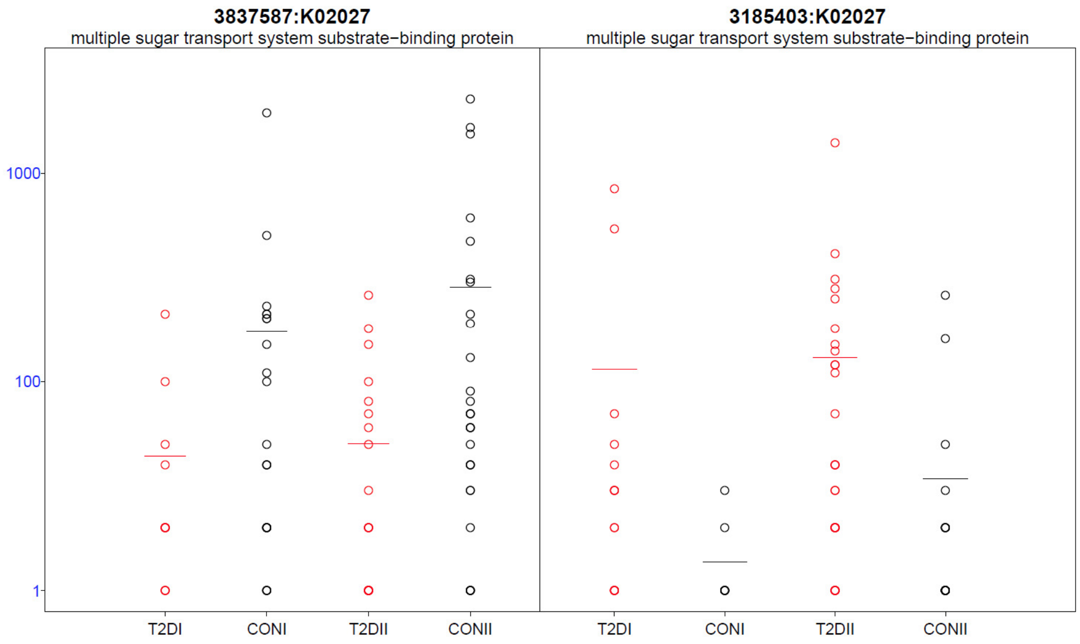

b

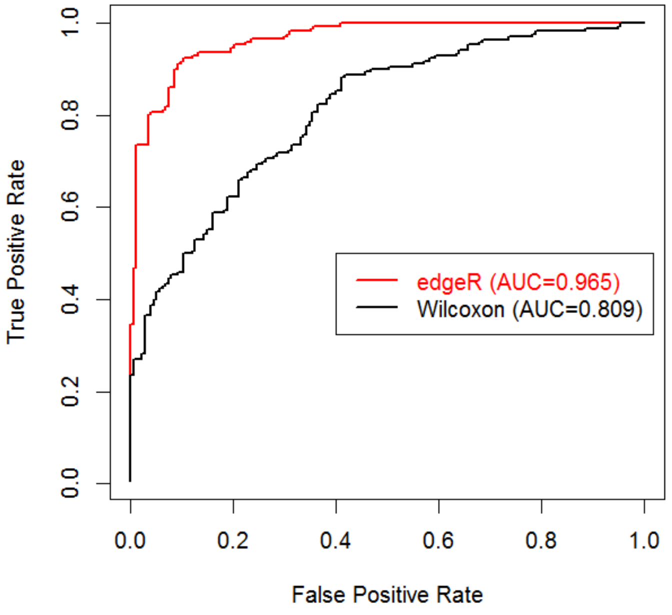

Supplementary Figure 11. Novel results from EDDA-guided analysis of gut microbiome data for patients with type 2 diabetes versus a control group. (a) Count distributions for novel Type 2 diabetes associated gut biomarkers. Count statistics for novel biomarker genes 3837587 and 3185403 are shown for cases (T2D) and controls (CON) in Stage I and Stage II datasets. (b) ROC curve for gut-microbiota-based patient classification (normal vs type 2 diabetes) using edgeR-derived and Wilcoxon-test-derived markers.

Dataset	Min	1st Quantile	Median	Mean	3rd Quantile	Max
Wu et al - Control	0.00	0.11	0.16	0.36	0.38	8.54
Wu et al – Case	0.00	0.13	0.21	0.45	0.48	8.84
hESC	0.017	0.03	0.05	0.12	0.09	3.33
MCF7	0.00	0.41	0.69	0.87	1.15	12.09
GM12892	0.00	0.09	0.19	0.33	0.43	2.99

Supplementary Table 1. Distribution of estimated sample variability across datasets. The table shows distributional statistics for dispersion values estimated by DESeq using different RNA-seq datasets.

	Stage I (p<0.05)	Stage II (p<0.01)	overlap	% of Stage I validated in stage II
**Wilcoxon**	221,454	112,977	33,410	15
**edgeR**	259,118	132,512	49,568	19

Supplementary Table 2. Differentially abundant genes discovered using the Wilcoxon test and edgeR in Stage I and Stage II datasets from Qin et al.

KO ID	# of genes	p-values	q-values	Description
**K02794**	23(21)	5.15E-06	0.017866063	PTS system, mannose-specific IIB component [EC:2.7.1.69]
**K03491**	30(23)	2.81E-05	0.017866063	lichenan operon transcriptional antiterminator
**K07718**	138(127)	2.75E-15	0.017866063	two-component system, sensor histidine kinase YesM [EC:2.7.13.3]
**K02026**	138(125)	6.19E-10	0.017866063	multiple sugar transport system permease protein
**K10117**	51(34)	4.98E-06	0.017866063	raffinose/stachyose/melibiose transport system substrate-binding protein
**K02025**	144(128)	1.23E-09	0.017866063	multiple sugar transport system permease protein
**K14051**	52(35)	6.15E-08	0.017866063	cyclic di-GMP phosphodiesterase Gmr [EC:3.1.4.52]
**K07720**	205(169)	1.90E-13	0.017866063	two-component system, response regulator YesN
**K02395**	12(12)	2.80E-05	0.017866063	flagellar protein FlgJ
**K11891**	9(9)	0.000301863	0.019106553	type VI secretion system protein ImpL
**K02795**	22(18)	0.00050654	0.020358069	PTS system, mannose-specific IIC component
**K02793**	20(18)	0.000747104	0.020358069	PTS system, mannose-specific IIA component [EC:2.7.1.69]
**K02083**	8(7)	0.000792672	0.020358069	allantoate deiminase [EC:3.5.3.9]
**K01143**	5(4)	0.001179103	0.023696458	exodeoxyribonuclease (lambda-induced) [EC:3.1.11.3]
**K02775**	13(9)	0.001590602	0.028098826	PTS system, galactitol-specific IIC component
**K13924**	24(18)	0.001659073	0.028196191	two-component system, chemotaxis family, CheB/CheR fusion protein [EC:2.1.1.80 3.1.1.61]
**K11688**	49(38)	0.001773889	0.028715814	C4-dicarboxylate-binding protein DctP
**K11904**	11(10)	0.00194088	0.028715814	type VI secretion system secreted protein VgrG
**K10254**	22(17)	0.002148737	0.028844504	myosin-crossreactive antigen
**K06859**	6(6)	0.002019187	0.028844504	glucose-6-phosphate isomerase, archaeal [EC:5.3.1.9]
**K10193**	11(8)	0.002818737	0.032549807	oligogalacturonide transport system permease protein
**K07497**	83(56)	0.003267791	0.036007585	putative transposase
**K02755**	27(18)	0.003534774	0.038094104	PTS system, beta-glucosides-specific IIA component [EC:2.7.1.69]
**K02950**	17(13)	0.003914817	0.041288766	small subunit ribosomal protein S12

Supplementary Table 3. List of top 20 novel gene families discovered using edgeR. The numbers in parentheses indicate the number of genes that are more abundant, on average, in type 2 diabetes patients. Enriched gene families (KEGG orthology groups, given by KO ids) were determined using Fisher's exact test with the differentially abundant genes identified using edgeR as input, and gene families not reported in Qin et al are reported here.

a

Marker	T2D	KO	Description
3263936	+	K00754	ko00051 Fructose and mannose metabolism
2056207	+	K00754	ko00051 Fructose and mannose metabolism
3609893	+	K01156	type III restriction enzyme [EC:3.1.21.5]
3274725	+	K02014	iron complex outermembrane recepter protein
3263912	+	K02042	phosphonate transport system permease protein
2776601	+	K02109	F-type H+-transporting ATPase subunit b [EC:3.6.3.14]
3385416	+	K02863	large subunit ribosomal protein L1
3393891	+	K02864	large subunit ribosomal protein L10
1372238	+	K03469	ribonuclease HI [EC:3.1.26.4]
3261825	+	K06994	putative drug exporter of the RND superfamily
2797559	+	K12983	UDP-glucose:(glucosyl)LPS beta-1,3-glucosyltransferase [EC:2.4.1.-]
2290296	-	K00595	precorrin-6Y C5,15-methyltransferase (decarboxylating) [EC:2.1.1.132]
4172153	-	K00820	glucosamine--fructose-6-phosphate aminotransferase (isomerizing) [EC:2.6.1.16]
4241215	-	K00936	E2.7.3.-
1401686	-	K01190	beta-galactosidase [EC:3.2.1.23]
3859391	-	K01235	alpha-glucuronidase [EC:3.2.1.139]
3655570	-	K01910	[citrate (pro-3S)-lyase] ligase [EC:6.2.1.22]
4240624	-	K01990	ABC-2 type transport system ATP-binding protein
3664159	-	K02115	F-type H+-transporting ATPase subunit gamma [EC:3.6.3.14]
3834139	-	K02172	bla regulator protein blaR1
347216	-	K02547	methicillin resistance protein
1235750	-	K02654	leader peptidase (prepilin peptidase) / N-methyltransferase [EC:3.4.23.43 2.1.1.-]
4186970	-	K02674	type IV pilus assembly protein PilY1
4226023	-	K03287	outer membrane factor, OMF family
3868905	-	K03327	multidrug resistance protein, MATE family
2429032	-	K03686	molecular chaperone DnaJ
3844995	-	K06969	23S rRNA (cytosine1962-C5)-methyltransferase [EC:2.1.1.191]
4264782	-	K07114	Ca-activated chloride channel homolog
4053911	-	K10742	DNA replication ATP-dependent helicase Dna2 [EC:3.6.4.12]

b

Marker	T2D	Marker	T2D	Marker	T2D
3451793	+	3852658	-	1337098	-
2861772	+	1846591	-	4048825	-
3541236	+	4171286	-	1146452	-
2841367	+	4146036	-	3951006	-
2924235	+	510823	-	4223515	-
3482270	+	3657490	-	3953310	-
2694357	+	4223528	-	4054373	-

Supplementary Table 4. List of marker genes identified by the mRMR feature selection approach. Marker genes with a known KEGG orthology (KO) based annotation are shown in (a) while those without are shown in (b). The T2D column indicates if the gene was found to be more ("+") or less ("-") abundant, on average, in patients with type 2 diabetes compared to normal controls.
