# Supplementary material for: The importance of study design for detecting differentially abundant features in high-throughput experiments
Source: Genome Biol. 2014 Dec 3;15(12):527. doi: 10.1186/s13059-014-0527-7 (PMC4253014; doi:10.1186/s13059-014-0527-7)
Supplement: Additional file 2: — Supplementary Text. [file 13059_2014_527_MOESM2_ESM.pdf]

How To: Add a new differential abundance test to EDDA

The following the step-by-step guide enables users to add new differential abundance tests to the EDDA package:

1. If it has not been installed yet, download and install the latest version of EDDA (https://sourceforge.net/projects/eddanorm/) and unzip the corresponding zip file into a directory (say EDDA_source).
2. Copy the .R file containing the R function for the new DAT (see **Code 1** for the Wilcoxon test) into the EDDA\R directory.
3. Modify "testDATs.R" to include the DAT as one of the many that can be called (see example code below in **Code 2** for adding the Wilcoxon test).
4. Modify "computeAUC.R", "plotROC.R" and "plotPRC.R" in a manner similar to the changes in "testDATs.R" (this is only necessary if the corresponding plotting functions are needed for the new DAT).
5. Re-build and install the modified package as before.

```
## R code for running the Wilcoxon test
run_wilcox_test <- function(counts, conds, cutoff){

  wilcox_test <- function(x) {
    x0=x[conds=="N"]
    x1=x[conds=="T"]
    pvalue=wilcox.test(x0,x1)$p.value
    if(is.na(pvalue)) {pvalue=1}
    return(pvalue)
  }

  all <- counts
  PValue <- apply(counts,1,wilcox_test)
  all <- cbind(all,PValue)
  result <- new("Result");
  result@data <- all[order(as.numeric(all$PValue)),];
  result@id <- rownames(result@data);
  result@pval <- result@data$PValue;
  return(result); # return all genes
```

Code 1. Wilcox.R

modify “testDATs.R” to add Wilcoxon test. The following is changes in testDATs() function (“……” means same as previous code and the **bold** part is adding part):

```
……
testDATs <- function(……
DE.methods=c("Cuffdiff",……,"NOISeq","Wilcoxon"), ……){
……
# add Wilcoxon result
  Result <-
list(……,MetaStats_nde=NULL,Wilcoxon=NULL,filterCounts=NULL)
……
# after Step 2.24 add one new job in analyses list
# Step 2.25: Running Wilcoxon analysis
    job_wilcox_test = function()

    if(is.element("Wilcoxon", method.list)==T){

      print("Starting Wilcoxon analysis...");
      Wilcoxon<-
tryCatch(run_wilcox_test(counts,conds,cutoff),
                         error=function(e) e);
      if(class(Wilcoxon)[1] != "Result"){

           print("Error running Wilcoxon:");
           print(Wilcoxon $message);
      }

      print("Wilcoxon analysis completed.");
      return(Wilcoxon);
}
……
result$MetaStats_nde <- parallel$job_MetaStats_nde;
[illegible]
```

Code 2. Modifications to testDATs.R to incorporate the Wilcoxon test. Note that “……” refers to unedited code that is omitted for brevity and text in **bold** are the modifications that are needed.

How To: Add new simulation model in EDDA

The following the step-by-step guide enables users to add new simulation models to the EDDA package:

1. If it has not been installed yet, download and install the latest version of EDDA (https://sourceforge.net/projects/eddanorm/) and unzip the
2. Modify "generateData.R" to add an additional simulation model as demonstrated in **Code 3** for the Poisson model.
3. Re-build and install the modified package as before.

```
……
generateData <- function(……){
……
   for(j in 1:EC){

     for(i in 1:(NR1+NR2))
       model.matrix[j,i] <- rnbinom(1, size=1/dispersions[j],
mu=model.rawFreq[j]*libsizes[i])

       if(model == "Poison")
          model.matrix[j,i] <-
             rpois(1,lambda=model.rawFreq[j]*libsizes[i])
   }
……
  if(model=="NegBinomial"||model=="Full"||model=="Poisson") {

    for(j in randomList) {

      coin <- coinList[index] # 0:up-regulation; 1:down-regulation
      fc <- fcList[index]
      for(i in 1:NR1){
      model.matrix[j,i] <- rnbinom(1, size = 1/dispersions[j],
                           mu=(coin*sqrt(fc)+(1coin)/sqrt(fc))
                           *model.rawFreq[j]*libsizes[i]);
       if(model == "Poisson")
          model.matrix[j,i]<-rpois(1,lambda=(coin*sqrt(fc)+(1-coin)
                            /sqrt(fc))*model.rawFreq[j]*libsizes[i])
      }

      for(i in (NR1+1):(NR1+NR2)){

         model.matrix[j,i] <- rnbinom(1,size=1/dispersions[j],mu=((
       1-coin)*sqrt(fc)+coin/sqrt(fc))*model.rawFreq[j]*libsizes[i])

         if(model == "Poison")
            model.matrix[j,i]<-rpois(1,Lambda=((1-coin)*sqrt(fc)
               +coin/ sqrt(fc))*model.rawFreq[j]*libsizes[i])
      `
```

Code 3. Modifications to generateData.R to add the Poisson model for simulation.
